# Naturally Acquired Antibodies against *Haemophilus influenzae* Type a in Aboriginal Adults, Canada

**DOI:** 10.3201/eid2102.140722

**Published:** 2015-02

**Authors:** Eli B. Nix, Kylie Williams, Andrew D. Cox, Frank St. Michael, Sandra Romero-Steiner, Daniel S. Schmidt, William G. McCready, Marina Ulanova

**Affiliations:** Northern Ontario School of Medicine, Thunder Bay, Ontario, Canada (E.-B. Nix, K. Williams, W.G. McCready, M. Ulanova);; National Research Council, Ottawa, Ontario, Canada (A.-D. Cox, F. St. Michael);; Centers for Disease Control and Prevention, Atlanta, Georgia, USA (S. Romero-Steiner, D.S. Schmidt)

**Keywords:** Antibody, IgM, IgG, bacteria, bactericidal, *Haemophilus influenzae* type a, Hia, polysaccharide antibodies, vaccine, indigenous, Aboriginal, Aborigine, antibody functional activity, secondary immunodeficiency, North America, Canada

## Abstract

In the post-*Haemophilus influenzae* type b (Hib) vaccine era that began in the 1980's, *H. influenzae* type a (Hia) emerged as a prominent cause of invasive disease in North American Aboriginal populations. To test whether a lack of naturally acquired antibodies may underlie increased rates of invasive Hia disease, we compared serum bactericidal activity against Hia and Hib and IgG and IgM against capsular polysaccharide between Canadian Aboriginal and non-Aboriginal healthy and immunocompromised adults. Both healthy and immunocompromised Aboriginal adults exhibited significantly higher bactericidal antibody titers against Hia than did non-Aboriginal adults (p = 0.042 and 0.045 respectively), with no difference in functional antibody activity against Hib. IgM concentrations against Hia were higher than IgG in most study groups; the inverse was true for antibody concentrations against Hib. Our results indicate that Aboriginal adults possess substantial serum bactericidal activity against Hia that is mostly due to IgM antibodies. The presence of sustained IgM against Hia suggests recent Hia exposure.

## Abstract 2

High prevalence of invasive Hia disease among North American Aboriginal populations is more likely related to exposure than to inadequate immunity.

*Haemophilus influenzae* is a human-restricted gram-negative bacterial pathogen that causes serious infectious diseases, including meningitis, sepsis, and pneumonia. Some strains express a polysaccharide capsule, a principal virulence factor that protects bacteria from immune defenses, e.g., complement-dependent bacteriolysis. On the basis of the chemical structure of the capsular polysaccharides, *H. influenzae* are divided into 6 serotypes (a, b, c, d, e, and f), and unencapsulated strains lacking the *cap* gene are referred to as nontypeable (*1*). *H. influenzae* type b (Hib) is the most virulent serotype; *H. influenzae* type a (Hia) is the second most virulent (*2*). Before the development of Hib conjugate vaccines in the 1980s, Hib was a major cause of pediatric meningitis (*3*). Hib conjugate vaccines induce production of antibodies against capsular polysaccharide capable of bactericidal activity providing protection against invasive disease (*4*–*6*). Rates of invasive Hib disease have been reduced by >90% in all countries where Hib vaccination programs have been introduced (reviewed in [*7*,*8*]).

Vaccination against Hib does not offer protection against other *H. influenzae* serotypes; in the post-Hib vaccine era, non–type b strains have become important in the etiology of invasive *H. influenzae* disease (reviewed by [*7*]). The course and severity of invasive Hia disease closely resemble the conditions caused by Hib (*9*). 

Although invasive Hia disease is rare in most of the world, it is prevalent in specific geographic areas including Northern and Western Canada, Alaska, and the southwestern United States; furthermore, the burden of disease falls almost exclusively on Indigenous peoples living within these regions (*10*–*12*). In the region of this study (Northern Ontario), invasive Hia disease was reported at rates of 7/100,000 in 2004–2008 and between 7.7–23.2/100,000 among children <5 years of age during 2002–2008 (*13*,*14*). Recent analysis of invasive *H. influenzae* disease, including Hia, in a population of Canada that included a large proportion of Aboriginal persons found that 54% of adult case-patients had some serious underlying medical conditions, such as chronic renal failure (CRF) (*13*). We hypothesized that a lack of naturally acquired antibodies against Hia may contribute to the higher rates of invasive Hia disease in this regional population. To test this hypothesis, we measured concentration of serum IgG and IgM against capsular polysaccharide and functional antibody activity against both Hia and Hib in healthy adults and patients of Aboriginal background with confirmed CRF. Measured antibodies in Aboriginal persons were compared to those present in serum samples from non-Aboriginal persons residing in the same region.

## Materials and Methods

### Study Subjects

We recruited a convenience sample of 70 Aboriginal and 70 non-Aboriginal healthy adults from the area surrounding Thunder Bay, Ontario, Canada. Health status was based upon self-assignment. There was no significant difference in age between the groups (Table 1). To study a population of immunocompromised adults, we determined that a sample size of 30 subjects/group would give a power of 85% with an α of 0.05 (2-sided *t* test) for detection of 2fold difference in mean geometrical titers of serum bactericidal activity against Hia. Therefore, we recruited 30 Aboriginal and 30 non-Aboriginal CRF patients undergoing hemodialysis at the Renal Services, Thunder Bay Regional Health Sciences Centre. On the basis of their age, 30 Aboriginal and 30 non-Aboriginal persons were selected for the healthy comparison group from the 140 persons in the healthy adult cohort to achieve a mean age that did not differ statistically from each ethnically matched CRF patient group (Table 1). None of the CRF or healthy comparison groups had been vaccinated against Hib. Among the 140 persons in the healthy adult cohort, 138 had not been vaccinated (we were unable to confirm Hib vaccination status of the remaining 2 persons). After acquiring informed consent from participants, we obtained serum samples and stored them at −80°C before use. All Aboriginal persons participating in the study were registered as such with the Canadian government. Recruitment took place during September 2010–August 2012. This study was approved by the Thunder Bay Regional Health Sciences Centre and Lakehead University Research Ethics Boards.

**Table 1:** Demographic characteristics of Aboriginal and non-Aboriginal groups studied for antibodies against *Haemophilus influenzae* type a, Thunder Bay region, northwestern Ontario, Canada, 2010–2012*

Group	No.	Age, y				No. (%) female	No. (%) ≥60 y
		Mean	Median	SD	Range		
Aboriginal CRF patients	30	54	54	±13.4	29–78	21 (70)	10 (33)
Aboriginal healthy comparison group	30	49.1	47	±9.4	39–75	25 (83)	4 (13)
Non-Aboriginal CRF patients	30	60.3	64.5	±13.9	26–79	11 (37)	20 (67)
Non-Aboriginal healthy comparison group	30	58.7	56	±9.4	45–80	18 (60)	11 (37)
Healthy Aboriginal adults	70	37.1	33.5	±12.7	19–75	58 (83)	4 (6)
Healthy non-Aboriginal adults	70	41.3	43	±14.1	22–68	44 (63)	7 (10)

*CRF, chronic renal failure. No significant age difference between healthy Aboriginal and non-Aboriginal adults (p = 0.067), Aboriginal and non-Aboriginal CRF patients (p = 0.081), Aboriginal CRF patients and Aboriginal healthy comparison group (p = 0.11), non-Aboriginal CRF patients and non-Aboriginal healthy comparison group (p = 0.60), p<0.05 between Aboriginal and non-Aboriginal healthy comparison groups (p = 0.0002). Healthy comparison groups (n = 30) were drawn from the large group of healthy adults (n = 70).

### Serum Bactericidal Assay

For the serum bactericidal assay (SBA), the strains used were Hia 08–191, isolated in 2008 from the blood of a 47-year-old Aboriginal man at Sioux Lookout, Ontario, Canada (*14*), and Hib strain 10–090, isolated in 2010 from the cerebrospinal fluid of a 1-year-old girl in Manitoba, Canada (provided by Dr. Tsang, the National Microbiology Laboratory, Winnipeg, Manitoba). The Hib strain had been previously used in SBA assays (*15*,*16*). The SBA was performed essentially as previously described for Hib (*16*–*18*), but in the Hia SBA, surviving bacteria were incubated for 17 h at 32°C to obtain clearly separated colonies for enumeration. The SBA titers were defined as the reciprocal serum dilution required to kill ≥50% of the initial bacterial inoculum as described by Rouphael et al. (*18*). The Hia SBA assay specificity had been previously determined to be 99.3% (*18*). Discontinuous titers below the lower detection limit of 8 were reported as 4 for statistical purposes.

### ELISAs for IgM and IgG against Hib Polysaccharide 

We determined serum polysaccharide IgG antibody concentrations against Hib using a *Haemophilus influenzae* type b IgG ELISA kit (IBL International, Hamburg, Germany) according to the manufacturer's instructions. For statistical analysis, concentrations below the lower limit of quantification were assigned half the lower limit of quantification. Serum IgM reactive against Hib polysaccharide was quantified as described (*15*). Serum IgG was depleted by mixing serum samples with IgG/RF stripper (The Binding Site, Birmingham, UK) (*15*). The standard curve was generated by using the reference serum FDA lot 1983 (*19*) after IgG depletion. The range of quantification was 0.017–3.5 μg/mL.

### ELISA for IgG against Hia Polysaccharide 

We developed the assay based on published methods with modifications (*20*). Following isolation, the polysaccharide was oxidized and conjugated to human serum albumin, then purified and characterized as described by Cox et al. (*21*). Hia polysaccharide conjugated to human serum albumin was coated into 96-well ELISA plates (final concentration 0.1 μg/well). As secondary antibody, horseradish peroxidase–conjugated mouse antibody against human IgG (Hybridoma Reagent Laboratory, Baltimore, MD, USA) was used in a 1:4,000 dilution. After the addition of Sure Blue TMB peroxidase substrate (Mandel Scientific, Guelph, Ontario, Canada) the colorimetric substrate was detected by using a microplate reader (BioTek Powerwave XS; Winooski, VT, USA) at 450 nm with 630 nm reference. Quantification of antibody was performed by using a previously described reference serum (4.1 μg/mL) as a standard (*20*). The quantification range was 0.10–4 μg/mL.

### ELISA for IgM against Hia Polysaccharide 

To quantify IgM against Hia polysaccharide, we used the protocol for IgG against Hia polysaccharide with modifications. After coating, the plates were blocked for 2 h at room temperature with antibody dilution buffer containing 1% fish gelatin (Sigma-Aldrich, Oakville, Ontario, Canada). Horseradish peroxidase–conjugated goat IgM against human IgM (SouthernBiotech, Birmingham, AL, USA) diluted 1:5,000 was used as the secondary antibody. The concentration of IgM against Hia polysaccharide (3.84 µg/mL) in the standard was determined by cross-standardization (*22*) to the Hib standard (Food and Drug Administration, 1983), which has an assignment of 3.5 μg/mL for IgM against Hib polysaccharide (*23*). The range of quantification was 0.01–18 μg/mL; for statistical purposes, samples with values less than the lower limit of quantification were assigned a value 1/2 the lower limit.

### Complement Activity

To rule out complement deficiency in study participants, we assessed the total classical complement pathway activity in serum samples using the CH_50_ Eq (Quidel, CA, USA) immunoassay according to the manufacture's protocol; results were expressed as CH_50_ equivalent units per mL.

## Statistical Analysis

We performed transformation of log_10_ data before analysis. Statistical significance was assessed by conducting Student *t* test using Graph-Pad Prism 5 (GraphPad Software Inc., San Diego, CA, USA). Geometric mean antibody concentrations (GMC), SBA geometric mean titers (GMT), and 95% CI were calculated for each group. The criterion of detectable SBA GMT was determined by using Fisher exact test; p values <0.05 were considered significant.

## Results

### Functional Antibody Activity Specific to Hia

Among a group of 140 healthy adults, Aboriginal persons had a significantly higher Hia SBA GMT, i.e., 351.4 (95% CI = 226.8–544.5) compared to the GMT of 182.8 (95% CI = 115.5–289.4) in the non-Aboriginal group (Figure). Similarly, Aboriginal CRF patients exhibited a significantly higher Hia SBA GMT than their non-Aboriginal counterparts, as shown in Table 2. There was a tendency toward lower Hia SBA GMTs in both Aboriginal and non-Aboriginal CRF-patient groups compared to their corresponding healthy comparison groups, but the differences were not statistically significant (Table 2).

**Figure:**
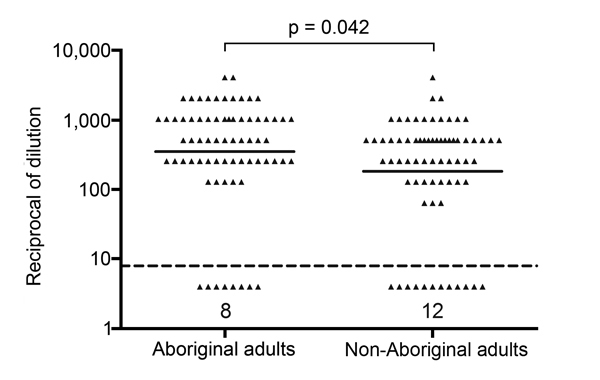
Antibody mediated bactericidal activity against *Haemophilus influenzae* type a in healthy Aboriginal (n = 70) and non-Aboriginal (n = 70) adults residing in the Thunder Bay region of northwestern Ontario, Canada, 2010–2012. The solid line indicates geometrical mean titer. The dashed line indicates the lower limit of detection; the number of individual samples below this limit is indicated on the graph.

**Table 2:** Concentrations of antibodies against *Haemophilus influenzae* type a and type b and serum bactericidal assay titers in Aboriginal and non-Aboriginal patients in chronic renal failure and comparison groups of healthy persons from the Thunder Bay region, northwestern Ontario, Canada, 2010–2012*

Variable	Age-matched comparison groups of healthy Aboriginal persons and those with CRF		Aboriginal CRF patients		Non-Aboriginal CRF patients		Non-Aboriginal healthy comparison group age-matched to non-Aboriginal CRF		Age-matched comparison group regardless of race		CRF patients regardless of race	
No. patients	30		30		30		30		60		60	
Hia SBA GMT	268.1		147.0		49.64		104.0		167.0		85.43	
95% CI	132.8–541.4		72.9–296.4		21.7–113.3		49.2–219.7		100–278.9		49.6–147.2	
p value†		p = 0.22		**p = 0.045**		p = 0.18				p = 0.076		
IgM against Hia, GMC	3.75		1.94		2.60		1.69		2.52		2.25	
95% CI	2.28–6.15		1.08–3.50		1.69–4.01		0.95–3.01		1.72–3.69		1.57–3.21	
p value		p = 0.086		p = 0.42		p = 0.23				p = 0.66		
IgG against Hia, GMC	1.81		1.56		1.20		1.27		1.52		1.37	
95% CI	1.26–2.60		1.12–2.17		0.89–1.63		0.91–1.77		1.19–1.93		1.10–1.71	
p value		p = 0.53		p = 0.25		p = 0.81				p = 0.54		
Hib SBA GMT	181.0		23.7		15.28		25.4		67.81		19.03	
95% CI	84.0–390.2		11.09–50.62		6.88–33.94		11.26–57.30		37.21–123.6		11.12–32.56	
P value		**p = 0.0003**		p = 0.42		p = 0.37				**p = 0.002**		
IgM against Hib, GMC	0.11		0.04		0.08		0.04		0.07		0.06	
95% CI	0.069–0.18		0.02–0.079		0.05–0.12		0.02–0.08		0.04–0.10		0.04–0.08	
p value		**p = 0.015**		p = 0.10		p = 0.089				p = 0.64		
IgG against Hib, GMC	1.63		0.61		0.62		0.87		1.19		0.61	
95% CI	1.06–2.51		0.39–0.95		0.34–1.11		0.61–1.24		0.91–1.58		0.43–0.88	
p value		**p = 0.0018**		p = 0.95		p = 0.31				**p = 0.004**		

*CRF, chronic renal failure; Hia* Haemophilus influenzae* type a; SBA, serum bactericidal assay; GMT, geometric mean titer; GMC, geometric mean concentration (μg/mL); CI, confidence interval; Hib, *Haemophilus influenza*e type b. †p values (Student *t* test [2-sided after log transformation]) are provided between each pairwise comparison. Significance level set at p<0.05; bold text indicates statistically significant p values.

### Serum Polysaccharide IgM and IgG against Hia

Aboriginal CRF patients showed a tendency to have lower concentrations of IgM against Hia polysaccharide than the Aboriginal healthy comparison group, but the difference was not statistically significant (Table 2). No difference in IgM against Hia polysaccharide was found between Aboriginal and non-Aboriginal CRF patients, non-Aboriginal CRF patients and the non-Aboriginal healthy comparison group, or between all CRF patients and all healthy comparison groups. Likewise, no difference in IgG against Hia polysaccharide concentrations between the groups was detected (Table 2). The combined IgM GMC against Hia of all groups was significantly higher (p = 0.0013) than IgG GMC against Hia, i.e., 2.38 μg/mL (95% CI = 1.84–3.08) versus 1.44 μg/mL (95% CI = 1.22–1.70); the average ratio of IgM against Hia to IgG against Hia was 1.65:1.

### Functional Antibody Activity Specific to Hib

No difference in serum bactericidal activity against Hib between Aboriginal and non-Aboriginal CRF patients was found. However, Aboriginal CRF patients had lower SBA GMT compared to the Aboriginal healthy comparison group. Overall, CRF patients had a lower SBA GMT than all healthy comparison groups (Table 2).

### Serum Polysaccharide IgM and IgG against Hib

Aboriginal CRF patients had significantly lower concentrations of IgM and IgG against Hib polysaccharide than the Aboriginal healthy comparison group. However, no statistically significant difference in IgM or IgG against Hib polysaccharides was found between Aboriginal and non-Aboriginal CRF patients, or non-Aboriginal CRF patients and non-Aboriginal healthy comparison group. Overall, CRF patients had a significantly lower IgG GMC compared to all healthy comparison groups (Table 2). The combined IgM GMC against Hib of all groups was significantly lower (p<0.0001) than IgG GMC against Hib, i.e., 0.061 μg/mL (95% CI = 0.045–0.082) versus 0.85 μg/mL (95% CI = 0.68–1.08); the average ratio of IgM against Hib to IgG against Hib was 0.072.

### Classical Complement Activity

The endogenous classical complement activity was measured in all study participants. The geometric means of all groups were in the range between 96.63 Eq U/mL (95% CI = 78.63–118.6) in the non-Aboriginal healthy comparison group and 118.9 Eq U/mL (95% CI = 99.13–142.5) in the Aboriginal healthy comparison group. The values were above the established cutoff point used to define abnormally low classical complement activity (<70 CH_50_ equivalent units per mL) according to manufacturer and did not statistically differ from each other (data not shown).

## Discussion

Compared to the general population, North American Indigenous populations have endured higher rates of invasive *H. influenzae* disease in both the pre- and post-Hib vaccine era (*12*). Although in the pre-Hib vaccine era, Hib was the major cause of invasive *H. influenzae* disease, cases attributed to Hia were also reported among some indigenous populations; for example, Hia accounted for 17% of invasive *H. influenzae* cases in a White Mountain Apache Indian community (October 1981–January 1983) (*24*). In the post-Hib vaccine era, Hia has emerged as a prominent cause of invasive disease among Alaska Natives and Aboriginal peoples of Canada (*14*,*25*,*26*). In northwestern Ontario (Canada), Hia is the serotype that most often cause invasive *H. influenzae* disease among Aboriginal persons, with an incidence rate of 7/100,000 during 2004–2008 (*13*,*14*). Recent studies in Northern Canada and Alaska found much higher prevalence of invasive Hia disease among the Aboriginal population than among the non-Aboriginal population. In Alaska, 88% of the cases occurred in Alaska Native persons (2002–2011) (*25*); in the Canadian North, 91% of the cases occurred in Aboriginal persons; the remaining 9% listed no ethnicity (2000–2010) (*26*). The incidence rate of invasive Hia disease in Alaska Native children <5 years in 2002–2011 was 36 × higher than in non-Native children (*25*). The factors related to an increased incidence of invasive Hia disease in Aboriginal populations are obscure. In this study, we questioned whether there were differences in the naturally acquired antibodies against Hia between Aboriginal and non-Aboriginal healthy and immunocompromised adults living in the same geographic area.

Our study shows an increased functional activity of serum antibody against Hia in Aboriginal adults of Canada than in non-Aboriginal persons living in the same area, which is likely caused by a substantial quantity of IgM antibodies. The presence of elevated IgM concentrations relative to IgG concentrations that are specific to Hia is suggestive of recent exposure to this pathogen in this study population. This is more apparent when the IgM against Hib were found to be low (<0.08 µg/mL) in most of the study groups, except for the Aboriginal healthy comparison group. These low levels of Hib antibodies can be attributed to the successful implementation of Hib vaccination (88% mean vaccine coverage) in the Canadian pediatric population and the effect of herd immunity (*27*).

In both healthy comparison groups and immunocompromised adults, IgM against Hia concentrations were higher than those of IgG, although the ratio was opposite for IgM and IgG isotypes of antibodies to Hib. A prevalence of IgM in the polysaccharide antibody repertoire against Hia among unvaccinated adults further suggests that naturally acquired antibodies could be the result of exposure to some cross-reactive antigens (*28*). High concentrations of IgM against Hib polysaccharide have also been found in nonvaccinated Alaska Native adults and potentially linked to continuous Hib carriage in this population (*23*).

Among healthy adults, serum antibody in the Aboriginal group exhibited much higher functional activity against Hia than that of the non-Aboriginal group as determined by SBA (Figure). Our findings contradict a presumption that a high rate of invasive Hia disease among Aboriginal peoples is due to a lack of naturally acquired antibodies. However, in northwestern Ontario, all adult cases of invasive Hia infection occurred in persons who had some underlying conditions associated with secondary immunodeficiency, such as type 2 diabetes or chronic obstructive pulmonary disease (*13*). Therefore, healthy adults may poorly represent the susceptible groups.

CRF patients undergoing hemodialysis are immunocompromised as a consequence of conditions underlying their renal failure as well as the hemodialysis procedure (*29*). Our previous research demonstrated that this patient group has a low concentration of antibodies against Hib that may be insufficient to protect against invasive Hib disease (*15*,*16*). Of note, Aboriginal peoples of Canada have a higher prevalence of end-stage renal disease and acquire it at younger ages than the general population (*30*). The SBA GMT against Hib was significantly lower in CRF patients than in healthy comparison groups which is consistent with our previous data on Hib immunity among patients in CRF (*15*,*16*). In addition to the presence of specific antibodies, the complement system is essential for defense against infections caused by gram-negative pathogens (*31*). Encapsulated *H. influenzae* are cleared by type specific antibodies against capsular polysaccharides, which activate the classical complement pathway leading to bacteriolysis (*32*). Because CRF patients often suffer protein loss related to the nephrotic syndrome and dialysis, their serum bactericidal activity in vivo may be impaired because of decreased complement concentration and lower antibodies in circulation. Because the SBA protocol uses a standardized exogenous complement source, a complement deficiency in tested serum samples would not influence the assay results. However, to rule out complement deficiency as a factor underlying susceptibility to invasive Hia disease, we tested the total classical complement activity in serum samples of study participants and found this parameter within the normal range in all the groups.

Although higher bactericidal activity against Hia was found in Aboriginal CRF patients than in non-Aboriginal ones, no significant difference in SBA GMT against Hib was detected between these groups. Despite major biological similarities between Hia and Hib, these 2 organisms have different natural histories because current vaccination programs cover Hib but not Hia. Pediatric Hib vaccination leads to a decreased carriage rate of Hib among adults by herd immunity; this may cause a decrease in natural boosting of antibody responses in nonvaccinated populations (*33*,*34*). Since there is no vaccine pressure on Hia, the difference in functional antibody activity between Aboriginal and non-Aboriginal adults may hypothetically depend on higher exposure to Hia in Aboriginal communities than in the general population. In addition, exposure to cross-reactive antigens of other bacteria, such as *Streptococcus pneumoniae* serotype 6B or *Bacillus pumilus* Sh 18 may potentially stimulate production of antibodies to Hia (*35*,*36*). Persistent circulation of *S. pneumoniae* in Aboriginal communities has been documented that may contribute to the development of natural immunity against Hia in Aboriginal adults (*37*). A review of the literature indicated only 1 other report of IgG measurements specific to Hia. Schmidt et al. analyzed an antibody against Hia in the cord blood of healthy neonates in Mexico and Chile; concentrations of IgG against Hia found in that study were 10- to 30-fold lower than the IgG against Hia in the CRF patients in this study (*20*). Because the predominant isotype of antibody against capsular polysaccharide is IgG2, which does not efficiently cross the placenta (*38*), analysis of neonatal cord blood samples may underestimate the quantity of antibody present in the maternal peripheral blood; cord blood would not include maternal IgM.

Our study has several limitations. In general, Aboriginal CRF patients undergoing hemodialysis are younger than their non-Aboriginal counterparts (*39*), and this was also true for our study groups. Therefore, a substantial age difference between the Aboriginal and non-Aboriginal healthy comparison groups precluded direct comparisons between the CRF groups and the large cohort groups. We compared each ethnic group to its own healthy comparison group to eliminate a possible effect of age on antibody concentrations and functional activity. Because children exhibit the majority of invasive Hia disease cases in our study region and elsewhere (*13*,*25*,*26*,*40*), the demographics of the present study do not fully reflect the immunoepidemiology of this infection. Future work should address natural immunity against Hia in the pediatric population. It will be important to study Hia antibody concentrations in neonatal cord blood serum samples in our population, as transplacentally acquired IgG may confer protection against invasive Hia disease in a similar manner to what is observed for Hib (*5*). Because the correlates of protection against invasive Hia disease have not yet been defined, the clinical interpretation of our findings is limited. Moreover, because of the sampling methods, this study may not represent other groups of North American indigenous peoples because they are highly genetically and phenotypically diverse and live in various environments. Extending the study of seroepidemiology of Hia infection will be critical to clarifying the reasons behind an increased susceptibility to Hia among certain population groups.

Although we did not conduct colonization studies, our results suggest that the high anti-Hia SBA titers in Aboriginal peoples are likely the result of a higher rate of Hia circulation within Aboriginal communities. It is crucial to study Hia colonization rates in this population to determine whether these natural antibodies are due to Hia exposure or exposure to cross-reacting bacterial species.
